# Therapeutic touch: influence on vital signs of newborns

**DOI:** 10.1590/S1679-45082013000400003

**Published:** 2013

**Authors:** Nadia Christina Oliveira Ramada, Fabiane de Amorim Almeida, Mariana Lucas da Rocha Cunha

**Affiliations:** 1 Hospital Israelita Albert Einstein, São Paulo, SP, Brazil.

**Keywords:** Therapeutic touch/nursing, Complementary therapies, Humanization of assistance, Intensive care, neonatal

## Abstract

**Objective>:**

To compare vital signs before and after the therapeutic touch observed in hospitalized newborns in neonatal intensive care unit.

**Methods:**

This was a quasi-experimental study performed at a neonatal intensive care unit of a municipal hospital, in the city of São Paulo (SP), Brazil. The sample included 40 newborns submitted to the therapeutic touch after a painful procedure. We evaluated the vital signs, such as heart and respiratory rates, temperature and pain intensity, before and after the therapeutic touch.

**Results:**

The majority of newborns were male (n=28; 70%), pre-term (n=19; 52%) and born from vaginal delivery (n=27; 67%). Respiratory distress was the main reason for hospital admission (n=16; 40%). There was a drop in all vital signs after therapeutic touch, particularly in pain score, which had a considerable reduction in the mean values, from 3.37 (SD=1.31) to 0 (SD=0.0). All differences found were statistically significant by the Wilcoxon test (p<0.05).

**Conclusion:**

The results showed that therapeutic touch promotes relaxation of the baby, favoring reduction in vital signs and, consequently in the basal metabolism rate.

## RESUMO

**Objetivo:**

Comparar os parâmetros vitais apresentados por recém-nascidos internados na unidade de terapia intensiva neonatal antes e após o toque terapêutico.

**Métodos:**

Trata-se de um estudo quase-experimental, de abordagem quantitativa, desenvolvido na unidade de terapia intensiva neonatal de um hospital municipal, na cidade de São Paulo (SP). A amostra constituiu-se de 40 recém-nascidos submetidos ao toque terapêutico após realização de procedimento doloroso. Foram avaliados parâmetros vitais, como frequência cardíaca e respiratória, temperatura e a intensidade da dor, antes e após o toque terapêutico.

**Resultados:**

A maioria dos recém-nascidos era do gênero masculino (n=28; 70%), pré-termo (n=19; 52%) e nascido de parto normal (n=27; 67%), sendo que o desconforto respiratório foi o principal motivo da internação (n=16; 40%). Houve queda de todos os parâmetros vitais após o toque terapêutico, principalmente do escore de dor – que apresentou redução considerável dos valores médios, de 3,37 (DP=1,31) para zero (DP=0,0). Todas as diferenças observadas foram estatisticamente significativas pelo teste de Wilcoxon (p<0,05).

**Conclusão:**

Os resultados evidenciam que o toque terapêutico promove o relaxamento do recém-nascido, favorecendo a redução dos parâmetros vitais e, consequentemente, a taxa de metabolismo basal.

## INTRODUCTION

Admission to the neonatal intensive care unit (NICU) places the newborn (NB) in a restricted environment, where he (she) is exposed to unpleasant stimuli, such as stress and pain. Noise, intense light, and the clinical and invasive procedures are constant, generating significant changes in the NB's vital signs^(1–3)^.

It is important to emphasize the need to provide assistance with a holistic approach, in such a way that care contributes to reducing the deleterious effects caused by hospitalization, both for NB as well as their family members^(4–6)^. The use of alternative measures, such as therapeutic touch, used to reestablish and rebalance body energy, can be very effective^(7–9)^.

Therapeutic touch is a healing method in which, by the use of hands, energy, warmth and love are transferred from a donor to the body of a recipient. This method guides the practitioner on how to proceed to concentrate and focus attention, a crucial part of every healing process^(9,10)^.

The practitioner then enters in harmony with the universal field, by means of a conscious interaction, so that they can guide the patient's vital energy in such a way as to reestablish their vitality^(5)^.

Besides the exchange of energy established between therapist and patient, the therapeutic touch, as well as other types of massage, also produces mechanic stimulation of the tissues, by means of applying pressure and elongation, compressing and putting tension in the soft tissues, and, as a consequence, stimulating the receptor nerve terminals^(11)^.

It is important to mention that skin pressure of different intensity gives origin to tactile stimuli, thus activating nerve receptors. This stimulus is converted into electrochemical reactions, which are sent to the posterior horn of the spinal cord and from there to the hypothalamus^(12)^.

Since the tactile fibers are more myelinated than the nervous ones (thick fibers), the tactile stimulus reaches the cord faster, inhibiting the thin fibers, which carry the painful stimulus. Besides that, when the hypothalamus is stimulated, endorphins and enkephalins are released and they have an action similar to morphine, acting over pain and generating a feeling of pleasure^(12)^.

Since the hypothalamus is the mediator system for emotions and regulates visceral functions, tactile stimulus may act over the autonomous system, relieving stress and anxiety^(12)^.

However, touching does not encompass only the physical aspect, but also an affective experience. This is due to the fact that nervous receptors, which are touch sensory elements, induce neurologic, glandular, muscle, and mental changes, which combined may produce or trigger emotions^(11)^.

Therapeutic touch is recognized as a nursing complementary practice by the Federal Board of Nursing (CFE, acronym in Portuguese), according to the COFEN resolution 197, of March 19, 1997, and it is allowed to be used in patient's care ^(13)^.

Like other complementary therapies, the therapeutic touch is an auxiliary treatment, not replacing conventional treatment, and should be done in parallel to the treatment plan proposed by the health professional team^(14)^.

A study using complementary application of therapeutic touch in women with breast cancer on chemotherapy demonstrated a reduction in side effects of the medication, such as nausea, vomiting, mucositis, anorexia, abdominal pain, esophagitis, diarrhea, and bowel constipation. This practice represented, therefore, an important tool to plan and implement nursing care to be dlivered by the nursing team to oncologic patients under chemotherapy^(15)^.

Another study addressed the use of massage as a means to grant comfort to women during labor, and demonstrated the therapeutic value of this procedure in promoting relaxation and relieving pain in that moment ^(16)^.

The application of therapeutic touch also proved beneficial to a polytrauma patient during his hospital stay, decreasing the intensity of “referred pain” by using the Pain Scale and, as a consequence, reducing the use of analgesics. It also led to improvement in arterial blood pressure and in the quality of sleep and rest^(17)^.

Considering the benefits reported in the literature in regard to the use of therapeutic touch, this study proposes to investigate its use as a way to give relief to stress experienced by neonates admitted to the NICU.

## OBJECTIVE

To compare vital signs of newborns admitted to neonatal intensive care before and after the application of therapeutic touch.

## METHODS

This is a quasi-experimental field study, of quantitative approach.

In the present study there was manipulation of variables, in this case the use of therapeutic touch (independent variable), and vital signs (dependent variable). The same group of NBs was assessed in regard to vital signs before (control group) and after (experimental group) the application of the therapeutic touch. Nevertheless, this choice was on purpose, since the first NBs admitted to the NICU during the data collection period were included in the sample.

The study was conducted at the NICU of the *Hospital Municipal Dr. Moysés Deutsch*, in São Paulo. The sample consisted of 40 NBs admitted to the NICU during October 2011, having their legal guardians agreed to their participation in the research, and signing the Informed Consent Form.

To collect the data, an instrument was designed encompassing information on the epidemiologic profile of the NBs and the vital signs before and after the application of therapeutic touch: temperature, heart and respiratory rates, and pain intensity.

The Neonatal Infant Pain Score (NIPS), specific for neonates, was used for pain assessment. It consists of five behavioral parameters and one physiological indicator evaluated before, during and after acute invasive procedures. Scores range from zero to 7, pain being considered for values ≥4^(18)^.

Data collection was initiated after the project approval by the Scientific Committee of the Nursing School of the *Hospital Israelita Albert Einstein* (HIAE), and by the Research Ethics Committee of the *Hospital Israelita Albert Einstein* (CEPHIAE) - CAAE: 0098.0.028.000-11, as well as after the authorization of the manager responsible for the unit where the data gathering took place.

Therapeutic touch was applied soon after painful procedures in the NB, and before starting it, a warm, well-ventilated and relaxing environment was provided, using reassuring background music at low volume ^(19)^.

The steps of the technique in NB are herein described: the baby was positioned comfortably while the investigator stood behind and to its side, maintaining the position of the hands in each region of the body for 3 minutes, in many parts, such as head, anterior and posterior chest, one at a time. Sessions lasted from 20 to 30 minutes, and further attention was dedicated to the ill parts of the body^(19,20)^.

Considering that the paired samples are dependent to evaluate the variation in NB vital signs after the therapeutic touch session, Wilcoxon signed rank test was used.

## RESULTS

The majority of the NBs who received the therapeutic touch were male (n=28; 70%), preterm (n=21; 52%) and born of vaginal delivery (n=27; 67%).

In relation to the reason for admission at the NICU, respiratory distress was the most frequent (n=16; 40%), followed by sepsis (n=13; 33%), and prematurity (n=7; 17%), among others.

The analyses of the NBs vital signs before and after the therapeutic touch session demonstrated that the mean values were lower after the session for all parameters, especially pain score, which had a considerable reduction - from 3.37 (DP=1.31) to zero (DP=0.0), as depicted on table 1.

**Table 1:** Vital signs presented by the newborns before and after therapeutic touch

Vital signs	Before		After		p value*
	Mean	Standard deviation	Mean	Standard deviation	
HR	138.6	27.3	116.4	15.6	0.000
RR	46.3	6.0	42.4	2.4	0.000
Temperature	36.4	0.5	36.2	0.3	0.049
Pain score	3.37	1.31	0.0	0.0	0.000

* Wilcoxon signed rank test: value statistically significant if p<0.05. HR: heart rate; RR: respiratory rate.

HR: heart rate; RR: respiratory rate.

**Figure 1:**
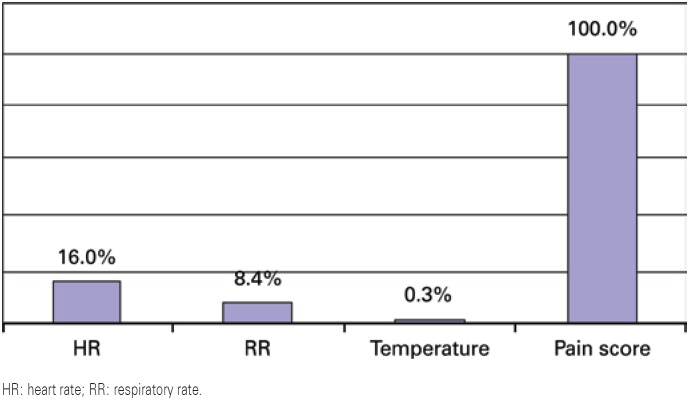
Percentage decrease in mean vital signs after therapeutic touch

All these differences observed were statistically significant according to the Wilcoxon test (p<0.05), demonstrating that effectively the therapeutic touch led to NB relaxation, when reducing basal metabolic rate, and therefore, the vital signs.

In this study, the significance level adopted was 5%, demonstrating statistically significant improvement in all four vital signs (p<0.05), as described on table 1.

Analyzing the average percentage of decrease in vital signs presented by the NB, the pain score presented the most marked drop (100%), followed by heart rate (16%).

## DISCUSSION

Therapeutic touch has been the subject of many studies and publications, as an alternative measure of comfort and well-being. The investigations aimed to demonstrate scientific evidence of its use correlate the touch mainly to reduced pain and stress and to induction of relaxation^(14,21,22)^.

The literature also points to the action of therapeutic touch in improving patients' responses to treatment, including wound healing and increase in hemoglobin levels in oncologic patients, even during chemotherapy, among other benefits^(10)^.

Concerning wound healing, an experimental study in guinea pigs submitted to provoked lesion demonstrated that those treated with water energized by means of therapeutic touch through placing hands, cured the lesions in a period of 20 days, which was superior to the control group, who received untreated water, and cure was observed in only 60% of the animals^(23)^.

These effects and many others are justified due to the close relation between the integumentary and nervous systems. In the embryonic phase, the epithelial and nervous systems are formed from cell differentiation from the ectoderm, a surface formed by cells that surround the whole embryonic body. Hence the functional complexity of the skin and its capacity as organ that modulates stimuli to the hypothalamic-pituitary-adrenal center^(12)^.

There is, therefore, continuous communication between the skin and the encephalus, which generates many events caused by endogenous hormones released in the blood flow and influences different systems in the body^(12)^.

In spite of the emphasis in drug treatment, the literature demonstrates that many non-pharmaceutical methods, such as changes in the hospital environment, making it a more adequate place for the child, decrease anxiety and fear, which may exacerbate pain. The following strategies are understood as non-pharmaceutical measures used to relieve pain and discomfort: tactile and kinesthetic stimulation, physical contact with the mother, non-nurturing suckling, musical therapy, reduction in environmental stimuli and skin-to-skin contact^(24)^.

It is therefore believed that the drop in vital signs after the therapeutic touch session may be related to tactile and kinesthetic stimulation of the baby.

A Brazilian study with 30 elderly patients that had the objective of evaluating the effect of therapeutic touch in chronic pain, in self-rating depression and sleep, also demonstrated significant reduction in pain (83.61%), decrease in the self-rating depression score (15.37%), and improvement in sleep patterns in these elderly subjects^(21)^. These data reinforce the ones found in the present study, in which the pain score presented by the NBs was also reduced after the use of this strategy, like other vital signs.

During data collection, the researchers of this study noted that the use of therapeutic touch in the NB also contributed to calm down the mothers, by noticing their children more relaxed after the application of the technique, especially after suffering a painful procedure. At the end of the session, the NB was really more relaxed, with calm face, sleeping for 1 or 2 hours, remaining calm after waking up.

The stimuli related to maternal care and from the environment are the mediators of the continuous neurobiological development of the NB and, above all, they determine the organization of the functioning neural networks, besides regulating the responses to the situations of pleasure and frustration^(25)^.

The results reinforce, once again, the importance of therapeutic touch as an effective strategy to render comfort to the NB, and at the same time, tranquility to the mothers, enabling them to more effectively participate in the child's care, since they also interact with their babies during the therapy.

This study corroborates the result of another research that used a technique with similar principles of placing hands and energy channeling - Reiki. This research verified that biological changes may be generated within a limited time frame, demonstrating the potential of this resource to improve clinically factors relevant to health status of the individuals^(22)^.

Although the results demonstrated effectiveness of therapeutic touch in decreasing vital signs, further studies with larger samples are needed to validate these findings.

## CONCLUSIONS

There were significant changes in all vital signs after the therapeutic touch, especially in pain score. The study demonstrated the effectiveness of therapeutic touch in the babies' relaxation and pain reduction.

Therapeutic touch is, therefore, a powerful humanizing strategy, since besides relieving pain and providing comfort to the NB, it fosters closer affective bonds between the child and their family members.
